# The impact of immigration and vaccination in reducing the incidence of hepatitis B in Catalonia (Spain)

**DOI:** 10.1186/1471-2458-12-614

**Published:** 2012-08-06

**Authors:** Manuel Oviedo, M Pilar Muñoz, Gloria Carmona, Eva Borrás, Joan Batalla, Nuria Soldevila, Angela Domínguez

**Affiliations:** 1 Department of Statistics and Operations Research, Universidade de Santiago de Compostela (St. Lope Gómez de Marzoa, s/n. Campus sur), Santiago de Compostela, 15782, Spain; 2 CIBER Epidemiología y Salud Pública (CIBERESP), Melchor Fernández Almagro 3-5, Barcelona, 28029, Spain; 3 Department of Statistics and Operations Research, Universitat Politècnica de Catalunya (UPC), Jordi Girona, 1-3, Barcelona, 08034, Spain; 4 Agency of Public Health of Catalonia, Roc Boronat 85, Barcelona, 08005, Spain; 5 Departament de Salut Pública, Universitat de Barcelona, Casanova 143, Barcelona, 08036, Spain

**Keywords:** Hepatitis B, Incidence, Vaccination, Immigration, GLM model, GAM model

## Abstract

**Background:**

The Hepatitis B virus (HBV) infection is a major cause of liver disease and liver cancer worldwide according to the World Health Organization. Following acute HBV infection, 1-5% of infected healthy adults and up to 90% of infected infants become chronic carriers and have an increased risk of cirrhosis and primary hepatocellular carcinoma. The aim of this study was to investigate the relationship between the reduction in acute hepatitis B incidence and the universal vaccination programme in preadolescents in Catalonia (Spain), taking population changes into account, and to construct a model to forecast the future incidence of cases that permits the best preventive strategy to be adopted.

**Methods:**

Reported acute hepatitis B incidence in Catalonia according to age, gender, vaccination coverage, percentage of immigrants and the year of report of cases was analysed. A statistical analysis was made using three models: generalized linear models (GLM) with Poisson or negative binomial distribution and a generalized additive model (GAM).

**Results:**

The higher the vaccination coverage, the lower the reported incidence of hepatitis B (p <0.01). In groups with vaccination coverage > 70%, the reduction in incidence was 2-fold higher than in groups with a coverage <70% (p <0.01). The increase in incidence was significantly-higher in groups with a high percentage of immigrants and more than 15% (p <0.01) in immigrant males of working age (19-49 years).

**Conclusions:**

The results of the adjusted models in this study confirm that the global incidence of hepatitis B has declined in Catalonia after the introduction of the universal preadolescent vaccination programme, but the incidence increased in male immigrants of working age. Given the potential severity of hepatitis B for the health of individuals and for the community, universal vaccination programmes should continue and programmes in risk groups, especially immigrants, should be strengthened.

## Background

The Hepatitis B virus (HBV) infection is a major cause of liver disease and liver cancer worldwide according to the World Health Organization. Following acute HBV infection, 15% of infected healthy adults and up to 90% of infected infants become chronic carriers and have an increased risk of cirrhosis and primary hepatocellular carcinoma. HBV is carried in the blood and other body fluid [1], including saliva, tears, semen and vaginal secretions, and person-to-person transmission is possible by various means, depending on the epidemiologic pattern within a geographic area [2]. In areas of high endemicity, the most common routes of infection are vertical mother-child transmission and horizontal transmission between children, particularly siblings. In areas of intermediate or low endemicity, such as Catalonia (Spain), the predominant modes of infection are sexual contact and intravenous drug use. Currently, hepatitis B is considered a major health problem, not only by health professionals, but also by many areas of society, especially sufferers [3] and the elevated health costs of the disease have been highlighted [4].

Safe, effective vaccines have been available to prevent hepatitis B virus infection since 1981 and the cost-effectiveness of hepatitis B vaccination programmes is well documented [5]. Recently, some recommendations on the identification and management of persons with chronic hepatitis B virus infection have been proposed [6,7], but vaccination is regarded as the best strategy to diminish the disease burden. In Catalonia, hepatitis B vaccination programmes began in 1984 with a vaccine obtained from human plasma that was expensive and had limited availability. Vaccination was carried out in groups at risk of suffering the disease due to lifestyles or work exposure and in newborns of mothers who were carriers of the virus, in accordance with the recommendations in place at that time. Unfortunately, this strategy had a limited impact on the incidence and long-term consequences of the disease, and highlighted the need for a strategy of universal vaccination in order to successfully prevent the disease. The availability of an effective, safe and cheaper vaccine obtained by genetic recombination opened the way for mass vaccination programmes to protect the whole population [8]. In December 1990, the Department of Health of Catalonia, after analyzing the available data on the impact obtained in terms of vaccination coverage and reduction of disease incidence with the vaccination programmes aimed at risk groups, decided to adopt a strategy of universal vaccination of preadolescents in schools, in addition to the vaccination of newborns from infected mothers and high risk groups [9]. In 2002, the Department of Health introduced the vaccination of newborns at 2, 4, and 6 months without abandoning the vaccination of preadolescents, which was of proven benefit [10]. This strategy, maintained until the first cohort of infants reached the age at which they could have been offered the preadolescent programme, seems to be the most comprehensive approach [1].

Infectious disease models have been proposed to estimate the burden of hepatitis B infection, and the impact of vaccination [11,12], in order to develop disease control strategies [13,14].

The aim of this study was to investigate the relationship between the reduction in acute hepatitis B incidence and the universal vaccination programme of preadolescents in Catalonia, taking population changes into account, and to construct a model to forecast the future incidence of cases that permits the best preventive strategy to be adopted.

## Methods

### Study population

The study was conducted in Catalonia, a region of more than 7 million inhabitants in Northeast Spain. All acute cases of hepatitis B reported to the Department of Health of the Generalitat of Catalonia from 1992 to 2007 (n = 2325) were included in the study. An acute case of hepatitis B was considered as an acute illness with a discrete onset of symptoms and jaundice or elevated serum aminotransferase that presented IgM against hepatitis B core antigen (anti-HBc) or hepatitis B surface antigen and negativity for markers of other hepatitis viruses. For the years 2005 to 2007, the country of origin of 74.2% of reported cases (n = 473) was known. In this period, 51% of the cases occurred in indigenous subjects and 49% in immigrants.

The study included the incidence of disease by age, gender, vaccination coverage, percentage of immigrants and year of report of the disease.

The population of Catalonia was obtained from the 2007 Catalan census (IDESCAT) [15]. The percentage of subjects born outside Spain was estimated by linear interpolation for the years in which data were not available (1992-1995 and 1997-1999).

Because the study was carried out with data reported by the Statutory Reporting System of the Department of the Generalitat of Catalonia without identification of the subjects, it was not necessary to be present it to the Ethics committee. All data used in this study are publicly available.

## Statistical methods

### Generalized linear model (GLM) and generalized additive model (GAM)

The statistical analysis was performed using the R statistical package, version 2.10.1 ( http://cran.r-project.org) Three models were used to adjust the incidence of hepatitis B: a generalized linear model (GLM) [16] with either Poisson or negative binomial distribution [17] and a generalized additive model (GAM) [18].

To determine which model best fit the incidence data, we estimated whether there was overdispersion in the data [19]. As the effect of continuous covariates may be non-linear, we made a nonparametric estimate using the smoothing techniques of the GAM model.

We made a new adjustment of the GLM and GAM models for the period 2000-2007 incorporating gender as a predictive variable. This was only possible from 2000 onwards, when the percentage of immigrants according to gender in the age group included in the study could be calculated.

The relationship between the response variable and the covariates was detected by studying the main effects and their interactions. The interactions were specified by taking the product of two main effects. The model includes interactions among the covariates. The best model was considered that with the minimum AIC (Akaike information criterion) and we compared the different adjusted models using the Anova test. Statistical significance was set assuming an α error = 0.05.

### Study variables

#### Dependent variables

Confirmed cases of hepatitis B in Catalonia *(cases).*

#### Independent variables or covariates

The covariates included in the model were: the year of report *(year),* vaccination coverage *(vac),* the percentage of the population born outside Spain *(immigrant)* and age *(age),* which was aggregated in six categories*:*

(1) $$age=\{\begin{array}{l} \begin{array}{ccc} ref & for & \begin{array}{ccc} age & <12 & years \end{array} \end{array}\\ \begin{array}{cc} 1 & \begin{array}{cc} for & 12\leq \begin{array}{ccc} age & <19 & years \end{array} \end{array} \end{array}\\ \begin{array}{cc} 2 & \begin{array}{cc} for & 19\leq \begin{array}{ccc} age & <34 & years \end{array} \end{array} \end{array}\\ \begin{array}{cc} 3 & \begin{array}{cc} for & 35\leq \begin{array}{ccc} age & <49 & years \end{array} \end{array} \end{array}\\ \begin{array}{cc} 4 & \begin{array}{cc} for & 50\leq \begin{array}{ccc} age & <59 & years \end{array} \end{array} \end{array}\\ \begin{array}{cc} 5 & \begin{array}{cc} for & \begin{array}{ccc} age & \geq 60 & years \end{array} \end{array} \end{array} \end{array}$$

### Adjusted models

All the models presented below contain the interaction, calculated as the product of two covariates; however they have not been included in the equations for the sake of simplicity.

Generalized linear models *glm.pois1* and *glm.nb1* (see eq.1) were used to estimate the incidence of hepatitis B in Catalonia adjusted by *year, age,vac, immigrant* and *population* as an offset parameter under the Poisson and negative binomial distributions, respectively.

(2) $$\log\left(cases\right)=\beta_{0}+\beta_{1}year+\beta_{2,\ldots ,6}age+\beta_{7}vac+\beta_{8}imnmigrant+\log\left(population\right)+\epsilon$$

The GAM *gam1 (see eq.2)* uses smoothing of the continuous variables *(year, vac* and *immigrant),* denoted by *s(year), s(vac)* and *s(immigrant),* respectively, and Poisson probability distribution, to adjust the incidence of hepatitis B:

(3) $$\log\left(cases\right)=\beta_{0}+\beta_{\mathrm{year}}s\left(year\right)+\beta_{2,\text{…6}}age+\beta_{\mathrm{var}}s\left(vac\right)+\beta_{\mathrm{immnimgrant}}s\left(immigrant\right)+\log\left(population\right)+\epsilon$$

The model in equation 1 was modified by substituting the variable *vac* by three categories*:*

(4) $$vac^{*}=\{\begin{array}{c} ref\;for\mathrm{cov}erage\;vaccination=0\%\\ 1\;for\;0<\mathrm{cov}erage\;vaccination<70\%\\ 2\;for\mathrm{cov}erage\;vaccination\geq 70\% \end{array}$$

and substituting the variable *immigrant* by four categories:

(5) $$immigrant^{*}=\{\begin{array}{l} \begin{array}{ccc} ref & for & \begin{array}{ccc} immigration & rate & <5\% \end{array} \end{array}\\ \begin{array}{cc} 1 & \begin{array}{cc} for & 5\%\leq \begin{array}{ccc} immigration & rate & <10\% \end{array} \end{array} \end{array}\\ \begin{array}{cc} 2 & \begin{array}{cc} for & 10\%\leq \begin{array}{ccc} immigration & rate & <15\% \end{array} \end{array} \end{array}\\ \begin{array}{cc} 3 & \begin{array}{cc} for & \begin{array}{ccc} immigration & rate & \geq 15\% \end{array} \end{array} \end{array} \end{array}$$

resulting in GLMs *glm.pois2* and *glm.nb2,* which were adjusted according to equation 3. The coefficients associated with the variables age, vac* and immigrant * in eq.3 are represented by a single parameter, but with several sub-indices, because these variables are categorical and they need as many coefficients as categories:

(6) $$\log\left(cases\right)=\beta_{0}+\beta_{1}year*+\beta_{2,\ldots ,6}age+\beta_{7,8}vac*+\beta_{9,10,11}immigrant*+\log\left(population\right)+\epsilon$$

Equation 4 shows the model for the years 2000-2007 in which the models from equation 1 were adjusted using Poisson and negative binomial, respectively, and the variable *gender* was also included. In this case, only the variables age and gender are categorical:

(7) $$gender=\{\begin{array}{l} \begin{array}{cc} ref & \begin{array}{cc} for & females \end{array} \end{array}\\ \begin{array}{cc} 1 & \begin{array}{cc} for & males \end{array} \end{array} \end{array}$$

(8) $$\log\left(cases\right)=\beta_{0}+\beta_{1}year^{*}+\beta_{2,\ldots ,6}age+\beta_{7}vac+\beta_{8}imnmigrant+\beta_{9}gender+\log\left(population\right)+\epsilon$$

Finally, we estimated the incidence of hepatitis B using the GAM gam1 * model in which continuous variables from equation 3 were adjusted by smoothing. Model gam2* was obtained by adding the estimate of the variable *gender* to model gam1* (see eq.5). In both cases, the probability distribution of errors was a Poisson distribution.

(9) $$\log\left(cases\right)=\beta_{0}+\beta_{\mathrm{year}}s\left(year\right)+\beta_{2,\ldots ,6}age+\beta_{\mathrm{var}}s\left(vac\right)+\beta_{\mathrm{inmnimgrant}}s\left(immigrant\right)+\beta_{9}gender+\log\left(population\right)+\epsilon$$

### Forecasting models and sensitivity analysis

The prediction of the incidence of hepatitis B was made using the adjusted models, and new data on the cova-riables, such as the number and percentage of immigrants according to age and sex, was necessary. The projections of these covariables were obtained from data provided by IDESCAT. However, the future percentage of the population vaccinated is not known *a priori,* and therefore various scenarios that allow different percentages of vaccination are necessary. For example, one possible scenario is to assume that the percentage of vaccination (variable *vac*) will increase by 5% each year. Other possible scenarios could be that the percentage of vaccination remains constant while supposing an increase in the incidence or in the proportion of immigrants in the age and sex groups, or that vaccination of some risk group could be considered. This study was concentrated on the prediction of vaccination in 2008 and 2009, assuming a constant annual increase of 5% in vaccination.

## Results

For the period 1992-2007, the estimated coefficients of the variables *year, age, vac* and *immigration* were significant in all adjusted models (eq.1, 2 and 3). Table 1 shows the results of the best adjusted generalized models, the GLM models using negative binomial distribution: *glm.nb1* and *glm.nb2*, and GAM *gam1*. In all models, the annual incidence decreased significantly (p-value <0.001). The best fit was achieved with the *gam1* model described in equation 2, (AIC = 593).

**Table 1:** Estimates of generalized linear models (glm.nb1 and glm.nb2) and additive models (gam1) for the study period 1992-2007

	**Model glm.nb1**		**Model gam1**		**Model glm.nb2**	
**Coefficients:**	**Estimate**	**Pr(>|z|)**	**Estimate**	**Pr(>|z|)**	**Estimate**	**Pr(>|z|)**
**Intercept**	136.99	<0.001	158.695	<0.001	80.771	<0.001
**Year (continuous**)	−0.075	<0.001	−0.085	<0.001	−0.046	<0.001
**Age < 12 y**	Ref.	-	Ref.	-	-Ref.	-
**12-18 y**	1.196	<0.001	1.291	<0.001	1.192	<0.001
**19-34 y**	1.515	<0.001	1.595	<0.001	1.721	<0.001
**35-49 y**	1.083	<0.001	1.076	<0.001	1.171	<0.001
**50-59 y**	1.024	<0.001	1.028	<0.001	0.987	<0.001
**> 59 y**	0.420	0.015	0.461	<0.001	0.335	0.015
**Vac (continuous**)	−1.926	<0.001	−2.297	<0.001	-	-
**Immigrant (continuous)**	6.404	<0.001	7.144	<0.001	-	-
**Vac = 0%**	-	-	-	-	Ref.	-
**0% < vac < 70%**	-	-	-	-	-.0700	<0.001
**vac > =70%**	-	-	-	-	−1.51	<0.001
**Immigrant < 5%**	-	-	-	-	Ref.	-
**5% < immigration < 10%**	-	-	-	-	0.159	0.307
**10% < immigration < 15%**	-	-	-	-	0.118	0.587
**immigration > 15%**	-	-	-	-	0.761	<0.001
**AIC**		622		593		625

**Figure 1:**
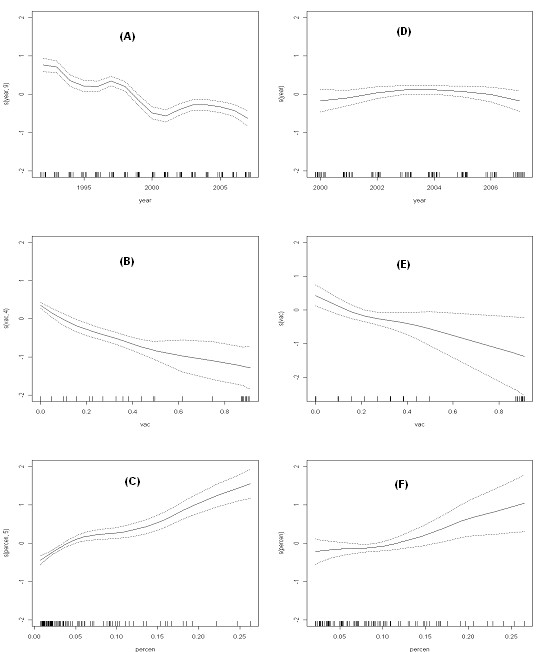
**The number of cases of hepatitis B have been modelled as smooth functions of year of report (*year*), vaccination coverage (*vac*) and proportion of immigrants (*percen*), using a GAM model.** Results for the gam1 model (period 1992-2007) are on the left panel and on the right panel, those for gam* model (period 2000-2007). The x axis of each plot is labelled with the covariate name and the y axis is labelled s(cov,edf) where cov is the covariate name, and edf the degrees of freedom of the smooth function. Upper and lower pointwise twice-standard-error curves are included (dashed lines).

Model *gam1* shows whether the trend of the variables was linear or not. Reporting of hepatitis B cases decreased over time until 2001, as indicated by the estimated smoothing coefficient of the variable *year* and its graphic representation (Figure 1A). From 2001 to 2004, there was an increase in the incidence of hepatitis B infection.

In the adjusted GLM models (*glm.nb1* and *glm.nb2* in Table 1) the incidence was higher in all age groups *(age)* for children under 12 years (p <0.001) but was less pronounced for people aged > 59 years (p = 0.015).

The percentage of vaccination was significant (p <0.001) in the *glm.nb1* model; the higher the rate of vaccination, the lower the incidence. In model *gam1*, vaccination coverage *(vac)* was introduced non-parametrically s*(vac)* and was significant (p <0.001). The trend was nonlinear and negative (Figure 1B), i.e., vaccination reduced cases of hepatitis B, and the greatest slope at the origin more clearly separates the vaccinated groups (with lower incidence) from unvaccinated groups. Model *glm.nb2* , which collected the categorization of vaccination coverage and the rate of immigration, can be considered as an alternative model to model *glm.nb1* (p <0.05), as confirmed by the ANOVA test, meaning there were no significant differences between models *glm.nb1* and *glm.nb2.*

Model *glm.nb2* shows that when the vaccination coverage was > 70% there was a two-fold higher reduction in incidence compared with vaccination coverages < 70% (Table 1). The opposite occurred with the percentage of immigration (model *gam1* ). The percentage of immigration showed a non-linear but positive trend (Figure 1C). The slope was steeper from a rate of 15% indicating that the greater the percentage of immigration, the greater the incidence. Groups with an immigration rate between 5% and 10% or between 10% and 15% did not have a greater incidence than when the percentage is < 5% (p = 0.307 and p = 0.587, respectively), while for a percentage of immigration of >15%, the increase in incidence was significantly higher than in groups with a percentage of immigration of<5% (p <0.001) (Table 1).

We identified the presence of four outliers, corresponding to the <12 years age group in 1998, 2001 and 2002, in whom the incidence was higher than estimated, and adolescents aged 12-18 years in 2000, with zero incidence. The residuals of the adjusted model were considered valid, and this model was proposed to explain and forecast the reported incidence of the disease.

**Figure 2:**
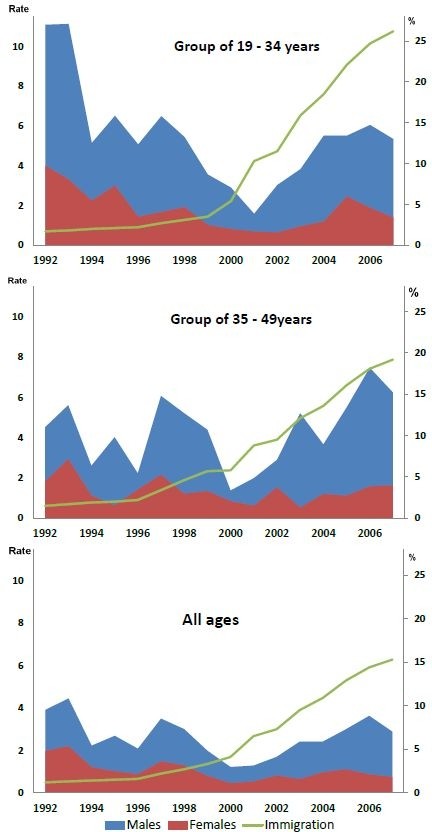
**Incidence rate by gender and percentage of immigrants in Catalonia.** Males in blue, females in red and % immigration in green.

Figure 2 panel (c) shows the temporal distribution of hepatitis B infection according to gender in the 19- 49 years age group (all ages group), which had the highest incidence, varying from > 8 x10^-5^ person-years in males and 3 x10^-5^ person-years in females in 1992 and 1993 to 1.8 x 10^-5^ person-years in males and 0.65 x10^-5^ person-years in females in 2001. However, from 2001 onwards, the incidence increased in males aged 19-49 years, reaching 6.7 x10^-5^ person-years in 2006, with a slight increase in females in the same age group to nearly 1.5 x10^-5^ person-years. The rate observed in the 19-34 years age group [Figure 2 panel (a)] declined until 2001 and then, despite the increase in vaccination coverage, rose again. The distribution of cases in the 35-49 years age [Figure 2, panel (b)] was similar from 2001 onwards, to the observed 19-34 years group. The proportion of immigrants increased over time (green line) and the increase was much greater from the year 2000 onwards.

The male/female ratio in incidence rates fluctuated between 3 and 4 x10^-5^ person-years, except in 2003, when six cases were reported in males aged 19-49 years for each case reported in women of the same age. The rates were less than for the total population, passing from 4 x 10^-5^ person-years in males and 2 x10^-5^ person-years in females in 1992 to 1.2 in males and 0.5 in females in 2001; in 2007 the rates rose to 3 x 10^-5^ person-years in males and 0.8 x10^-5^ person-years in females. The male/female ratio increased over time, from 2 reported cases reported in males for each case in females in 1992 to almost 4 reported cases in males for each case in females in 2007.

Incidence rates for children aged < 12 years were below 2 x10^-5^ person-years and there were 4 cases, all immigrants, in the last year, 2007. As vaccination coverages rose in the 12-18 years age group, the reported incidence rate fell until 1995 and remained low (<1 x10^-5^ person-years) until 2007.

The variable *gender* was incorporated into the regression models for the period 2000-2007, when the proportion of immigrants could be calculated according to *gender*, *year* and *age*. Table 2 shows the results of different models with and without the inclusion of the variable *gender*. Once again, the models with negative binomial distribution had a lower AIC and therefore a better estimate than the models which assumed a Poisson distribution. The inclusion of the variable *gender* always improved estimates (ANOVA test: p <0.001). Model glm.nb1* (eq.1) had an AIC of 554, while the model using negative binomial distribution including *gender* had an AIC of 485 *(*model *glm.nb3*, eq.4). In the GAM models, the AIC passed from 604 in model gam1* (eq.2) to 487 in model gam2 * (eq.5). Models glm.nb3* and gam2* showed similar results: in both cases the estimate of the variable *year* was not significant (p > 0.05). As shown in Figure 1D, smoothing by year in the gam2 * model can be assimilated to the trend remaining approximately constant throughout the study period.

**Table 2:** Estimates of models glm.nb1*, glm.nb3*, gam1* y gam2* for the study period 2000-2007

	Model glm.nb1*		Model glm.nb3*		Model gam1*		Model gam2*	
**Coefficients:**	**Estimate**	**Pr(>|z|)**	**Estimate**	**Pr(>|z|)**	**Estimate**	**Pr(>|z|)**	**Estimate**	**Pr(>|z|)**
**Intercept**	245.03	0.024	82.95	0.280	376.20	<0.001	−7.39	0.916
**Year (continuous**)	−0.13	0.018	−0.05	0.214	−0.19	<0.001	−0.002	0.941
**Age < 12 y**	Ref.	-	Ref.	-	-	-	Ref.	-
** 12-18 y**	2.95	0.0267	1.37	0.074	4.96	<0.001	1.70	0.011
** 19-34 y**	0.84	0.039	0.87	<0.001	1.28	<0.001	1.27	<0.001
** 35-49 y**	0.15	0.627	0.56	0.015	−0.013	0.945	0.76	<0.001
** 50-59Y**	0.96	<0.001	0.91	<0.001	1.01	<0.001	0.84	<0.001
** > 59Y**	0.57	0.039	0.35	0.09	0.70	<0.001	0.09	0.612
**Vac (continuous**)	−3.80	0.016	−1.74	0.050	−6.40	<0.001	−2.03	0.009
**Immigrant (continuous**)	3.57	<0.001	8.11	<0.01	20.39	<0.001	5.44	0.008
**Gender**	-	-	0.88	<0.01	-	-	0.89	<0.001
**AIC**		554		485		604		487

The significance of the percentage of vaccination was greater for model gam2* than for model glm.nb3* (p = 0.009 and p = 0.05, respectively), and the trend remained negative and nonlinear (Figure 1E). The percentage of immigration was significant in both models (p <0.01) and the trend remained positive and nonlinear (Figure 1F). The inclusion of gender in the models allowed a better fit, and the estimated coefficients of the variable *gender* in both models were similar and significant (p <0.01 and p <0.001 respectively, Table 2).

The residuals for the GLM and GAM models for the period 2000-2007 (eq. 1, 4, 2 and 5, respectively) were also considered correct. Interactions between *gender* and other covariates (*age*, and/or *year*, *vac* or *immigration*) were not significant.

**Figure 3:**
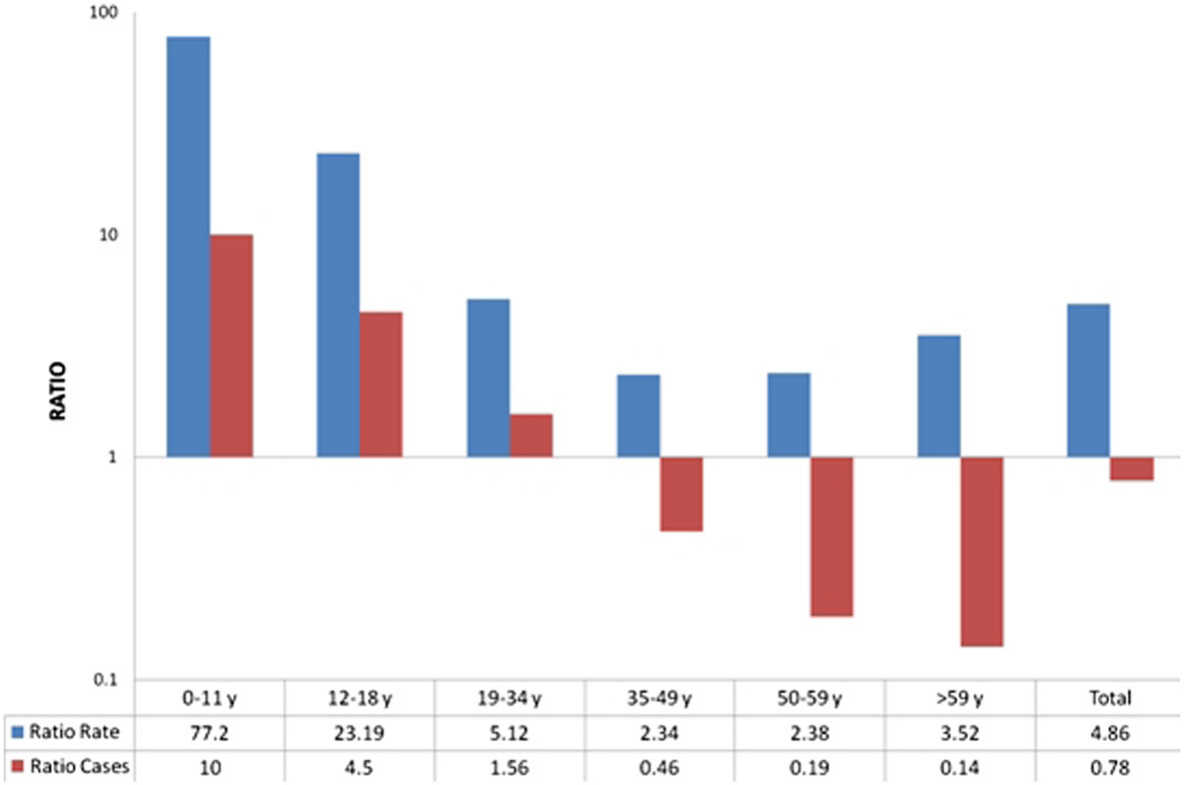
Ratio (immigrant/indigenous) of the incidence rate per 100,000 population per year and cases of hepatitis B in Catalonia 2005-2007.

The percentage of cases that occurred in immigrants during the 2005-2007 period decreased with age; 91% in immigrant children aged < 12 years, 82% in immigrants aged 12-18 years, 61% in young adults and only 14.5% in immigrants aged > 50 years. As shown in Figure 3, this means that the ratio between the incidence rate in immigrants and in indigenous persons was higher in children aged < 12 years (ratio = 77.2, rate of 3.86 x10^-5^ person-years in immigrant children compared to 0.05 x10^-5^ person-years in indigenous children) and in people aged 12- 18 years (ratio = 23.19, rate of 3.7 x10^-5^ person-years compared to 0.2 x10^-5^ person-years in indigenous children).

**Figure 4:**
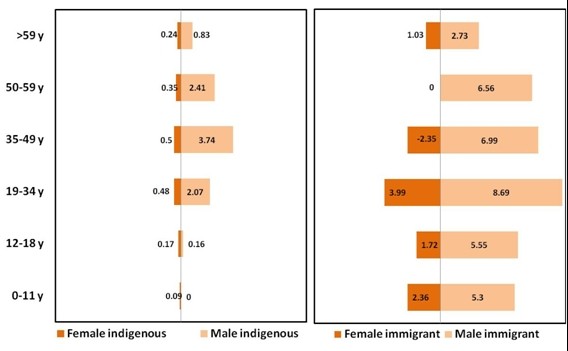
Incidence rate in Catalonia 2005-2007 according to: age group (y axis), gender (dark orange) for females and (pale orange) for males and nationality (left panel) for indigenous subjects and (right panel) immigrants.

Figure 4 shows the distribution of the incidence in immigrants according to gender and age group for the period 2005-2007. The incidence rate for male immigrants was > 5 in all age groups except the > 59 years age group. In people aged < 18 years, the origin (born in Spain or outside) was known in 18 of the 21 cases (86%). In immigrant girls, the incidence rate was around 2.36 x10^-5^ person-years and in boys it was more than double, at 5.3 x10^-5^ person-years (p <0.001). The incidence in indigenous children aged < 19 years was <0.2 person-years in both males and females, but was 5.55 x10^-5^ person-years in immigrant males aged < 19 years, much higher than the nearly 2 x10^-5^ person-years in immigrant females of the same age (p <0.001).

Tables 3 and 4 show the results of the prediction for the models described in Tables 1 and 2, respectively, assuming a constant increase of 5% in vaccination in 2008 and 2009. The gam models were clearly the best models for the prediction, as they were those which best adjust the non-lineal behaviour of the temporal evolution of the incidence of hepatitis B. The gam2 model (Table 3) estimated incidence rates of 1.76 (95% CI: 1.62-1.90) and 1.54 (95% CI: 1.41-1.67) for 2008 and 2009, respectively, compared with the 1.68 and 1.63 observed in 2008 and 2009, respectively. In 2008 (Table 4) the gam2* model estimated an incidence rate of 0.98 (95% CI: 0.87-1.11) compared with the observed rate of 1.00: likewise, the estimate was 0.89 (95% CI: 0.78-1.011) in 2009, compared with an observed rate of 0.93. In males, the incidence rate is overestimated in 2008 (3.03 (95% CI: 2.72-3.38)) compared with the observed rate of 2.38. In 2009, the observed rate was 2.38, compared with the estimated rate of 2.69 (95% CI: 2.38-3.04). As in the estimate, the prediction is better when the variable *gender* is included in the model, although it is slightly worse for the gam1* model and better results are obtained for the gam2* model.

**Table 3:** **Incidence predicted and 95% confidence predicted interval (CI) by year for models in Table**1

**Year**	**Incidence Observed**	**Incidence predicted by model glm.nb1**	**Incidence predicted by model gam1**	**Incidence predicted by gam2**
2008	1.68	1.94 (1.69-2.39)	1.88 (1.64-2.15)	1.76 (1.62-1.90)
2009	1.63	1.94 (1.66-2.26)	1.79 (1.56-2.05)	1.54 (1.41-1.67)

**Table 4:** **Incidence predicted and 95% confidence predicted interval (CI) by year and gender for models in Table**2

**Year**	**Gender**	**Incidence Observed**	Incidence predicted by model glm.nb1*	Incidence predicted by model glm.nb3*	Incidence predicted by gam1*	Incidence predicted by gam2*
	Females	1.00	2.06 (1.68-2.53)	1.31 (1.16-1.47)	1.33 (1.17-1.50)	0.98 (0.87-1.11)
2008	Males	2.38	3.27 (2.61-4.11)	4.08 (3.65-4.59)	3.98 (3.55-4.46)	3.03 (2.72-3.38)
	All	1.68	2.66 (2.14-3.31)	2.68 (2.40-3.01)	2.65 (2.36-2.97)	2.00 (1.79-2.24)
	Females	0.93	2.17 (1.73-2.71)	1.37 (1.21-1.57)	1.40 (1.23-1.60)	0.89 (0.78-1.01)
2009	Males	2.34	3.43 (2.68-4.40)	4.27 (3.78-4.83)	4.19 (3.69-4.76)	2.69 (2.38-3.04)
	All	1.63	2.80 (2.02-3.55)	2.81 (2.48-3.19)	2.79 (2.45-3.17)	1.78 (1.57-2.02)

## Discussion

As with any vaccine-preventable disease, hepatitis B vaccination programmes should be reviewed according to the evolution of the disease, and surveillance data are crucial to enable correct assessments of the situation [1].

The results of our study are consistent with those for hepatitis A, which showed that the estimates of GLM using negative binomial distribution were better [19]. In addition, GAM permitted estimation of non-linear trends of continuous variables as *year, vac* and *immigrant.* The incidence of hepatitis B fell from 1992 to 2000, and has since increased non-linearly, being much higher in groups with a high percentage of immigration and falling when vaccination coverages are higher.

The results also coincide in the case of categorical variables in the GLM models. Poisson or negative binomial regression can cause problems in estimating the coefficients due to the low incidence of cases. This is a limitation of the model that was solved by aggregation into age groups, and not introducing it as a continuous variable. An alternative would have been to adjust using models for count data with Poisson distribution with many zeros (ZIP) or negative binomial (ZINB) [20].

With respect to the predictions, future studies could simulate the predictions of future incidence rates with vaccination strategies other than those proposed in this study. This could allow evaluation of these strategies and the detection of atypical incidence rates other than the estimated pattern.

The use of two data sets (1992-2007) and (2000-2007, which included the category male/female, has advantages and disadvantages. Using the (1992-2007) database has the advantage of a 16-year historical record in which the estimated coefficient of the variable *year* is significant. This is one possible explanation of why the estimates are better using this database. In the second case, the use of the (2000-2007) database supposes a historical record of only eight years. The positive aspect is that the clearly-differentiated incidence rates in males and females can be estimated. However, the estimated coefficient of the variable *year* is not significant in this period in the models with the lower AIC (glm.nb3 y gam2). As the number of observations is reduced, the number of degrees of freedom and, therefore, the estimates, are worse.

La Torre et al. [12], applied jointpoint regression and estimated that the incidence decreased in all groups, and highlighted the importance of analyzing the changes in disease incidence in the evaluation of vaccination policies.

The increased incidence in men aged 19-49 years from 2001 onwards obtained in the present study with the overall rate increasing from 2.16 in 2000 to 5.74 in 2007(x10^-5^ person-years) respectively, coincides with the mass influx of immigrants to Catalonia with rose from 5.6% in 2000 to 22.7% in 2007 [15]; in this same period, the incidence in men aged 19-34 years increased from 2.9 in 2000 to 5.3 in 2007 (x10^-5^ person-years) and in men aged 35-49 years from 1.4 in 2000 to 7.5 in 2007 (x10^-5^ person-years). These results differ from those obtained in the USA [21,22], where the incidence rate of hepatitis B has decreased steadily in all age groups, from 6.3 x 10^-5^ person-years in 1992 to 1.5 x10^-5^ person-years in 2007. In that country, although progress has been made in reducing disparities in incidence of new infections rates among non-Hispanic blacks have declined, the incidence rates remain more than twofold higher than those among other ethnic population and the rate in elderly immigrants is much lower than in young adults, although the number of elderly immigrants is very low. Several authors have already analyzed the relationship between the incidence of hepatitis B and immigration, showing that there is a positive relationship between both [23,24].

In 1992, the rate in females in Catalonia was 2 x10^-5^ person-years, which fell to 0.45 x10^-5^ person-years in 2000 and remained fairly stable in the remainder of the study period. In the USA [22] the rate fell from 7.34 in males and 5.01 in females (x10^-5^ person-years) in 1992 to 3.6 and 2.09 x 10^-5^ person-years, in males and females, respectively, in 2000 and 1.85 in males and 1.15 in females (x10^-5^ person-years) in 2007, while in Germany, rates fell from 1.96 x10^-5^ person-years in 2001 to 0.89 x10^-5^ person-years in 2006 and 0.6 x10^-5^ person-years in 2007.

In people aged 12-18 years, the incidence was zero in 2000 and less than one case per year, later. No gender differences in the incidence were detected in indigenous children of vaccination age or in children aged < 12 years, with very low or zero rates. The rates in indigenous women and people aged < 18 years were less than 1x10^-5^ person-years, as a result of the vaccination programs implemented. In contrast, in immigrants and other age groups, the reported incidence was more than two-fold higher for men than for women.

The male-female ratio doubled over the study period, unlike the results in the USA [22] and Germany [25]. In the USA, the male-female ratio increased slightly, from 1.5 in 1992 to 1.8 in 2006, and in Germany, it rose from 1.91 in 2001 to 2.2 in 2008. This may be explained by the increased incidence in immigrants, a group containing a large proportion of men of working age. The statistical models used show that the incidence of cases increased due to immigration, especially in groups with > 15% of immigrants.

Although the impact of disease prevention measures and the maintenance of high vaccination coverages are important, this impact may be offset by an increase in cases in adult immigrants. The proportion of immigrants aged >50 years is still low, which could explain the low incidence of acute hepatitis B in this population compared to younger ages. Therefore, as suggested by the distribution of cases in other countries [22,25], the incidence may increase in these groups. For this reason, vaccination strategies for risk groups, including travellers to countries with high or intermediate prevalence of chronic Hepatitis B virus infection should be reinforced [26,27]. This recommendations should also be applicable to susceptible immigrants (children and adolescents) coming from countries with high or intermediate prevalence where hepatitis B vaccination programmes have still not been launched or where coverages are still very low [28].

In countries like the USA, Germany and Italy, incidence rates have declined in all age groups. In Germany, the rate for males decreased from 2.8 x10^-5^ person-years in 2001 to 1.2 x10^-5^ person-years in 2007, while in the USA [22] the rate fell from 3.6 and 2.09 (x 10^-5^ person-years) in 2000 to 1.85 and 1.15 (x10^-5^ person-years in 2007 in males and females, respectively. In Catalonia, the rate increased from 1.3 x 10^-5^ person-years in 2001 to 2.9 x10^-5^ person-years in 2007. However, it should be noted that, in Germany, the percentage of immigrants has remained constant at around 9% since 1995 [25]. In the USA the percentage of immigrants increased from 10.4% in 2000 to 12.6% in 2007 [29]. However, in Catalonia, the rise was much higher, from 4.1% in 2000 to 15.7% in 2007.

This study was conducted using routine surveillance data. It would probably be useful to focus more-closely on immigrants and risk groups to obtain a better understanding of the situation of hepatitis B virus infection in order to design strategies to increase vaccination coverages [21,30].

In addition to the protection afforded to individuals in avoiding the risk of chronic liver diseases such as cirrhosis and hepatocellular carcinoma [31], a strategy focused not only on universal vaccination of infants or preadolescents but on risk groups, and immigrants should be considered because it takes into account the substantial indirect effects of vaccination, as avoiding new infections avoid the cases transmitted by them [32].

A possible limitation of this study is the underdetection of cases in the immigrant population. The attendance of health services is free in Catalonia for all people (indigenous and immigrants), but we do not know if there are differences in the attendance to medical services for acute hepatitis B. So, we cannot rule out some underdetection of cases in immigrant population. In order to improve our estimates we would need to improve the surveillance of acute hepatitis B disease and to know what level of completeness of reporting we have for immigrant and indigenous population.

With respect to the quality of the proposed forecasts, the randomness in the incidence pattern of the disease is much lower than in males, making it much easier to predict the temporal disease evolution. However, the randomness in the incidence is greater in males, depending, amongst other factors, on age and disease outbreaks, and therefore it is more difficult to predict its future evolution. The observed incidence was lower than the confidence intervals of the predictions, indicating a change in the pattern of the evolution of the disease.

Future research might concentrate on extending the proposed models to include the spatial-temporal distribution of the disease, as we have done for hepatitis A [33]. Likewise, Pearce and Dorling [34] studied a period of rapid social, economic and political changes in which differences in life expectancy between men and women had a clear geographical dependence. It is essential that future studies have access to more reliable information on immigration and that they can recover missing data whenever possible, in order to detect disease outbreaks earlier and adjust the vaccination strategy to these situations.

## Conclusions

In conclusion, the results of the models used in this study confirm that the overall incidence of hepatitis B has declined in Catalonia due to the universal vaccination of preadolescents, but that the incidence has increased in male immigrants. Given the seriousness of hepatitis B virus infection for individuals and for the community, universal vaccination programmes should be continued and measures to increase vaccination of risk groups and immigrants, should be reinforced.

## Competing interests

The authors declare that they have no competing interests.

## Authors’ contribution

All the authors participated in the design, implementation and interpretation of the study. MO and GC had full access to all the study data and take responsibility for the data and accuracy of the data analysis, MO, MPM and AD designed the study and drafted the report; EB and JB contributed to obtain and supervised all information about vaccines, MO, MPM and NS conducted the statistical analysis and contributed to editing the manuscript. All authors read and approved the final manuscript.

## Pre-publication history

The pre-publication history for this paper can be accessed here:

http://www.biomedcentral.com/1471-2458/12/614/prepub
